# The Role of ISL1 and LHX5 LIM Homeobox Genes in Bladder Tumourigenesis

**DOI:** 10.21315/mjms2020.27.1.4

**Published:** 2020-02-27

**Authors:** Mohd Khairul Anuar Md Akhir, Chan Soon Choy, Maizaton Atmadini Abdullah, Fauzah Abd Ghani, Abhi Veerakumarasivam, Huzlinda Hussin

**Affiliations:** 1 Department of Pathology, Faculty of Medicine and Health Sciences, Universiti Putra Malaysia, Selangor, Malaysia; 2 Perdana University School of Foundation Studies, Perdana University, Selangor, Malaysia; 3 Department of Biological Sciences, School of Science and Technology, Sunway University, Selangor, Malaysia; 4 Malaysia Genome Institute, National Institute of Biotechnology Malaysia, Selangor, Malaysia; 5 Medical Genetics Laboratory, Faculty of Medicine and Health Sciences, Universiti Putra Malaysia, Selangor, Malaysia

**Keywords:** bladder cancer, LIM homeobox, tumourigenesis

## Abstract

**Introduction:**

Lin-11, Isl-1 and Mec-3 domains (LIM) homeobox genes are among the most important sub-families of homeobox genes. These genes are thought to play an important role in cancer. In this study, the protein expression of these genes was examined in urothelial carcinoma of the bladder. The expression pattern of Islet-1 (ISL1) and LIM homeobox 5 (LHX5) across different cancer stages and grades, as well as the association between the protein expression of these genes and patient demographics and clinicopathological features, were examined.

**Methods:**

A total of 100 formalin-fixed paraffin-embedded urothelial carcinoma tissues were selected from the Department of Pathology, Hospital Kuala Lumpur and the protein expression of ISL1 and LHX5 was determined using immunohistochemistry.

**Results:**

Positive expression of ISL1 and LHX5 was detected in 94% and 98% of the samples, respectively. There were no distinct LHX5 expression patterns associated with different cancer stages, but the proportion of high-expressing tumours was higher in high-grade tumours. In addition, there was a significant association between the expression of LHX5 and tumour grade. The proportion of tumours expressing high levels of ISL1 was found to be highest in later stage tumours.

**Conclusion:**

The high percentage of tumours expressing both these genes suggests that ISL1 and LHX5 play an important role in bladder tumourigenesis across multiple stages.

## Introduction

Lin-11, Isl-1 and Mec-3 domains (LIM) homeobox genes are among the most important sub-families of homeobox genes. They encode a series of LIM-homeodomain (LIM-HD) proteins featuring two LIM domains in their amino termini and a centrally located HD that is used to interact with specific DNA elements in target genes (1, 2). There are at least 12 LIM homeobox genes in humans and they encode key regulators of the developmental pathway. Recently, studies have shown that LIM homeobox genes also play an important role in cancer and 10 LIM-HD proteins have been reported to be associated with various types of cancer (2).

Islet-1 (ISL1) and LIM homeobox 5 (LHX5) belong to the sub-family of LIM homeobox genes. ISL1 plays an important role in various cellular processes, such as cytoskeleton genesis, organogenesis and oncogenesis (3). It has been found to be a highly specific marker for pancreatic endocrine tumours and metastasis (4, 5). High expression of ISL1 is also associated with the depth of tumour invasion, lymph node metastasis, the histological grade of the tumour and poor survival in gastric cancer patients (6). In addition, ISL1 promoter methylation is frequently found in bladder cancer. An increase in ISL1 promoter methylation is significantly associated with increased tumour number, size, grade and stage in bladder cancer (7). It is also significantly correlated with aggressive tumour characteristics, tumour recurrence, progression and disease-specific mortality (DSM) in bladder cancer (7, 8).

LHX5 has been found to play an essential role in the regulation of neuronal differentiation and migration during the development of the central nervous system (9). It is also essential for the regulation of precursor cell proliferation, neuronal differentiation and migration during hippocampal development in mice. LHX5 has been reported to have prognostic value in breast cancer (2). To date, there have been few studies characterising the role of LHX5 in cancer.

Urothelial carcinoma is the 10th leading cause of death worldwide (10). It is also highly recurrent. About 70% of superficial urothelial carcinomas (Ta and T1) will recur after the first treatment and 10%–20% of them will progress to invasive urothelial carcinoma (11, 12). Thus, frequent and long-term surveillance is needed for the management and treatment of the disease. This causes a massive economic burden to patients and governments, making it the most expensive cancer (per patient) to manage. Because previous studies have indicated that these two proteins play a potential role in cancer, this study aimed to characterise the protein expression of these genes in a cohort of formalin-fixed paraffin-embedded tissues from patients with urothelial carcinoma of the bladder.

## Methods

### Sample and Data Retrieval

All demographic factors and clinicopathological parameters of urothelial carcinoma patients were retrieved from the Department of Pathology and Records Unit of Hospital Kuala Lumpur, Malaysia. One hundred histological blocks of paraffin-embedded tissue of urothelial carcinoma cases collected between 2008 and March 2013, and representative of diverse tumour stages and grades were obtained from the Department of Pathology at Hospital Kuala Lumpur. All urothelial carcinoma samples were obtained from biopsy, cystectomy, cystoprostatectomy and cystectomy plus total abdominal hysterectomy with bilateral salpingo-oophorectomy (TAHBSO) specimens. Other types of carcinoma and unsatisfactory samples, such as large areas of tumour necrosis, were excluded from this study. Haematoxylin and eosin (H&E) stained slides of all the samples were reviewed independently by two pathologists. Patients’ demographic data and clinicopathological parameters (age, gender, race, muscle invasiveness, tumour stage and grade, lymph node involvement and metastasis) were tabulated.

### Sectioning and Preparation of Slides

Four-micrometre segments of each paraffin block were sectioned and placed in a water bath containing distilled water at 40 °C. The sectioned tissues were then fixed on the poly-L-lysine slide (Thermo Scientific, USA). The slides were then stored at 4 °C.

### Immunohistochemical Staining

#### Control tissue

Tissues from normal testes were used as a positive control for both antibodies tested. Matched negative controls were stained without the primary antibody.

#### Hydration and antigen retrieval

The slides were deparaffinised via incubation at 60 °C for 45 min in an oven, VENTICELL (MMM Group, Germany) followed by soaking into two xylene solutions for 5 min. The slides were then rehydrated by soaking them in a sequence of 100%, 100%, 95%, 80% and 70% ethanol for 5 min each. Then the slides were washed with running tap water for 5 min. Antigen retrieval was performed with a citrate buffer (10 mM, pH6) on high mode for 5 min to boil, followed by a defrosting mode for 10 min in the oven (ELBA, Republic of Korea). The slides were cooled at room temperature for about 35 min. A circle was drawn onto the glass slides surrounding the tissue sample using a Pap pen (Daiko Sangyo, Japan) before washing the slides with TBS plus Tween 20 solution (TBST-20) 5 times for 2 min each time. The slides were then blocked with 150 μL of 3% hydrogen peroxide for 30 min.

#### Antibody incubation

The slides were then washed with TBS-T 20 solutions for five times, 2 min each time. Hundred microlitres of primary antibody (Table 1) was added onto the slides.

#### Antigen detection

The slides were then washed with TBST-20 solutions 5 times for 2 min each time before adding four drops of polymer (DAKO REAL^™^ EnVision^™^/HRP) onto the slides and then incubated for 30 min. The slides were washed again with TBST-20 solutions 5 times for 2 min each time. Next, 100 μL of Chromogen diaminobenzidine (1 of Dako REAL^™^DAB: 50 Chromogen of Dako REAl^™^ substrate buffer) was applied onto the slides for 5 min. The slides were then rinsed in running tap water for 10 min.

#### Counterstain and dehydration

The slides were then counterstained with Hematoxylin-Z (CellPath, UK) for 2 min and then rinsed again under running tap water for 10 min. Slides were dehydrated by soaking in a sequence of 70%, 80%, 95% and 100% ethanol solution for 3 min each. The slides were then soaked in xylene solution for 5 min twice and subsequently mounted with Dibutylphthalate Polystyrene Xylene (DPX). Upon completion of the staining procedure, the slides were observed under a light microscope for scoring.

#### Scoring for immunohistochemical staining

The immunostained slides were semi-quantitatively scored by two independent pathologists. The slides were scored based on the percentage of cells that were positive in staining and the intensity of the staining (Table 2). The final score for each slide was obtained by multiplying the percentage of positivity and the staining intensity score. Zero score was interpreted as negative (non-expressing tumours), scores that were less than or equal to 4 (≤ 4) and more than or equal to (≥ 6) were interpreted as low- and high-expressing tumours, respectively (13). In instances where there were discrepancy in the slides scoring, the slides were reviewed and scored together by the pathologists to reach a consensus agreement.

### Statistical Analysis

Statistical analysis was performed using SPSS 16.0 software package for Windows. The association between ISL1 and LHX5 expression with the demographic and clinicopathological parameters as well as the correlation between ISL1 and LHX5 expression were analysed using Chi-squared test. A *P*-value < 0.05 was considered as statistically significant.

## Results

### Immunohistochemical Expression and Localisation of ISL1 and LHX5 in Urothelial Carcinoma

**Figure 1:**
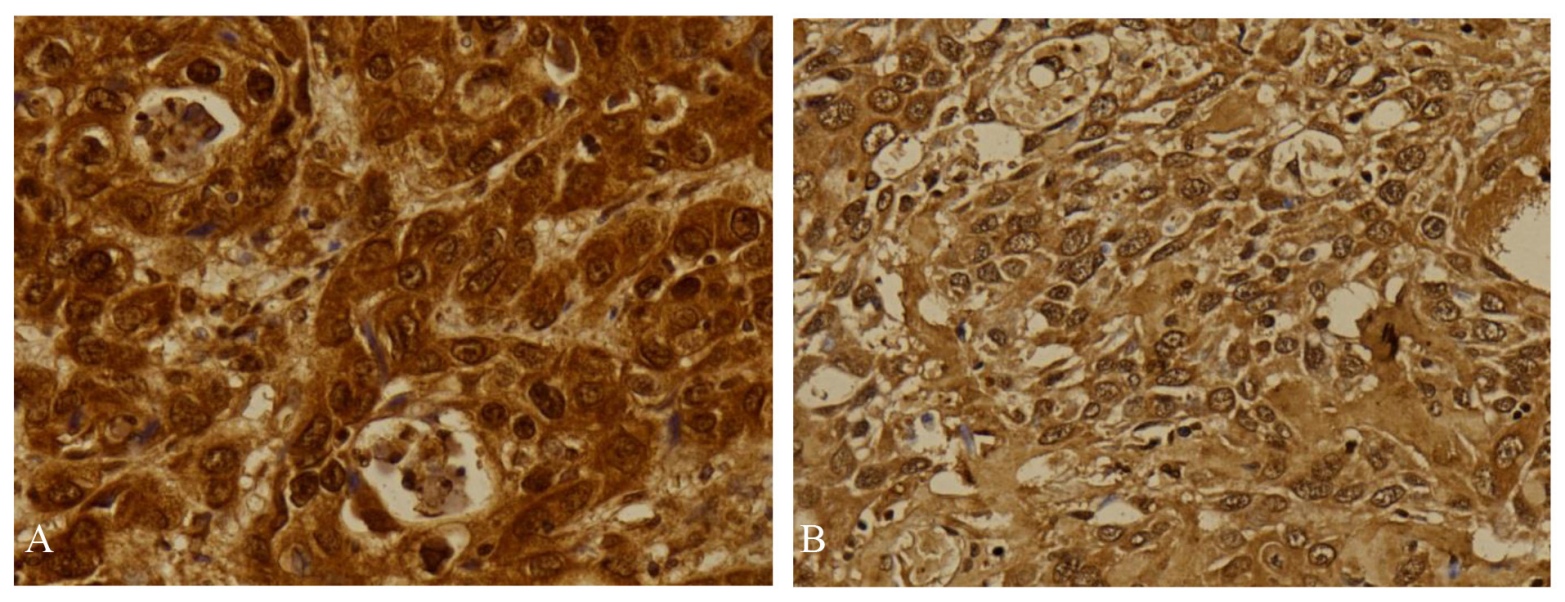
Immunohistochemical staining of ISL1 in urothelial carcinoma showing high expression (A) and low expression (B) (400×)

Most of the samples showed positive expression for both ISL1 and LHX5 proteins. For ISL1, 94 out of 100 samples (94%) showed positive immunohistochemical staining. High expression of ISL1 was found in 67 (71.3%) samples and the remaining 27 (28.7%) samples showed low expression (Figure 1). Thirty-two (34%) of the stained samples displayed nuclear staining. Both nuclear and cytoplasmic staining were observed in 30 (31.9%) samples, while 29 (30.9%) samples showed cytoplasmic staining only. Only 3 (3.2%) samples showed nuclear membrane staining.

**Figure 2:**
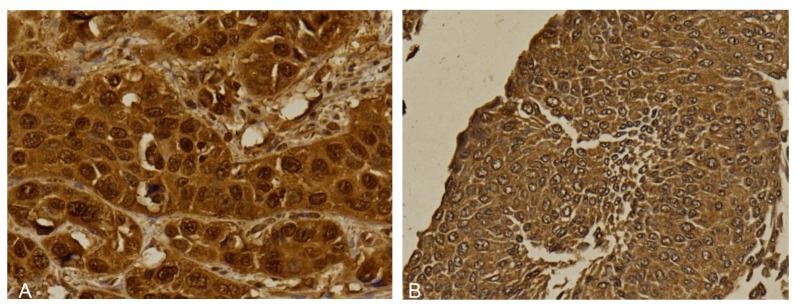
Immunohistochemical staining of LHX5 in urothelial carcinoma showing high expression (A) and low expression (B) (400×)

Positive LHX5 expression was found in 98 (98%) of samples. Of which, 75 (76.5%) samples showed high expression and the remaining 23 (23.5%) showed low expression of LHX5 (Figure 2). LHX5 was expressed in both nucleus and cytoplasm in the majority of samples (52; 53.1%). Twenty-eight (28.6%) samples showed cytoplasmic staining only while 14 (14.3%) samples showed nuclear staining only. Only 4 (4.1%) showed nuclear membrane LHX5 staining.

### Immunohistochemical Expression of ISL1 and LHX5 Across Different Stages and Grades of Urothelial Carcinoma of the Bladder

The percentage of high ISL-expressing tumours was highest in stage 4 tumours. In fact, there was an increase in the percentage of high ISL-expressing tumours as tumour stage progressed from 1 to 4. In contrast, the percentage of high ISL-expressing tumours reduced as tumour grade increased. Nevertheless, ISL was expressed highly in more than 60% of tumours across all tumour stages and grades (Table 3).

In contrast, there was no distinct trends observed in the expression of LHX5 based on the tumour stage and grade. The highest percentage of high LHX5-expressing tumours were stage 1 tumours and grade 2 tumours. With the exception of stage 3 tumours (53.8%), high LHX-expressing tumours were found in more than 75% of tumours across all tumour stages (Table 3).

### The Association of ISL1 and LHX5 Protein Expression with Demographic and Clinicopathological Parameters of Urothelial Carcinoma of the Bladder

The association between ISL1 and LHX5 expression with demographic factors and clinicopathological parameters were statistically tested using Chi-squared test. Among the demographic factors that were included were gender (male or female), age (< 50 or ≥ 50) and race (Malay, Chinese, Indian and others). The clinicopathological parameters that were included were stage (stage 0, stage 1, stage 2, stage 3 and stage 4), grade (low grade and high grade), muscle invasiveness [non-muscle invasive bladder cancer (NMIBC) and muscle invasive bladder cancer (MIBC)], lymph node metastasis and distant metastasis.

There were no significant associations between the expression of ISL1 with the demographic and clinicopathological features tested (Table 3). However, there was a significant association between the expression of LHX5 with the tumour grade (low/high grade) of urothelial carcinoma of the bladder. There was a greater percentage of high LHX5-expressing tumours in high grade tumours as compared to low grade tumours. However, there were no significant associations between LHX5 immunohistochemical expression and other demographic and clinicopathological features (Table 3).

## Discussion

Immunohistochemistry is a semi-quantitative method that requires a few considerations regarding antibody selection, antibody concentration and tissue fixation (14). Nevertheless, it is an effective method to characterise the expression and localisation of proteins and provide an assessment of the potential utility of putative biomarkers (15–17). In general, the immunohistochemical protein expression characteristics of ISL1 and LHX5 reflect their potential roles in urothelial carcinoma of the bladder tumourigenesis.

The sub-cellular localisation of proteins in specific tissue determines their function in the cells (18). Nuclear staining of ISL1 was in concordance with previous studies that analysed the endocrine cells of pancreatic islets and neuroendocrine tumours (4, 19). Although there were no previous studies that examined the expression of LHX5 in cancer tissues, one study conducted in rats, discovered nuclear staining of LHX5 in cajal-retzius cells; the first neuronal cell type in mammalian telencephalon (20).

ISL1 and LHX5 are classified in the same homeobox family, LIM-HD family. They are then further sub-grouped into the LHX family (3). LHX family proteins usually act as transcription factors or cofactors and they are mainly localised in the nucleus (3, 21). Transcription factors play an important role in the production of mRNA before they are translated into protein. The transcription process takes place in the nucleus of the cell. However, the localisation of these two proteins in the nuclear membrane was unexpected. It would be interesting to investigate further the biological significance of the translocation of these proteins in the nuclear membrane.

ISL1 and LHX5 expression was found across all tumour grades and stages of urothelial carcinoma of the bladder. ISL1 was previously found to play a role in the regulation of the cytoskeleton, organogenesis as well as oncogenesis. It was also found to be a good marker for pancreatic neuroendocrine tumours (19). ISL1 also promotes the proliferation of non-hodgkin lymphoma cells in vitro (22). There was a study that identified a significant correlation between the methylation of ISL1 with increasing stage and grade of bladder cancer; suggesting that expression levels reduced as tumour progressed (8). A recent study by Kitchen et al. (7), in 2015 also showed that ISL1 was highly methylated in high-grade tumours as compared to lower or intermediate grade tumours. Our study showed decreasing ISL1 expression as the grade of the urothelial carcinoma of the bladder increased which supports the methylation data of previous studies. Not many studies have been conducted on LHX5 in cancer. Studies done by Miquelajáuregui et al. (20), in mice show that LHX5 is involved in the control of cell differentiation in the telencephalon region rather than in proliferation. The present study is the first study characterising LHX5 protein in urothelial carcinoma of the bladder. In this study, there was a lower proportion of high LHX5-expressing tumours in stage 3 and stage 4 tumours as compared to stage 1 and stage 2 tumours; perhaps indicating a more pronounced role during the earlier stages of tumourigenesis.

The association of these two proteins with demographic and clinicopathological parameters of urothelial carcinoma patients was conducted to evaluate the relationship between their expression and patient prognosis. ISL1 was a transcription factor that possesses two N-terminal LIM domains and one C-terminal homeodomain. LIM domains are protein structural domains that mediate protein-protein interactions critical to cellular processes. ISL1 expression was recently found to show significant correlation with the depth of tumour invasion, lymph node metastasis and TNM stage of gastric cancer (6). A study on pancreatic endocrine tumour also showed significant correlation between ISL1 expression and tumour metastasis (4). To date, there are still no studies done for ISL1 in advanced stages of bladder cancer tissue especially in bladder cancer that have metastasised. We did not find any significant correlation between the expression of ISL1 with demographic and clinicopathological features. This might be because different types of cancers are regulated by different orchestrated and stochastic transcriptomic and genetic changes during tumourigenesis (23).

LHX5 expression is significantly associated with the tumour grade (low/high grade) of urothelial carcinoma of the bladder. A previous study reported that LHX5 was found to have prognostic value; independent of the sub-types and other clinical factors in breast cancer (2, 24). However, this study did not find any significant correlations between LHX5 expression and the various demographic factors and other clinicopathological parameters tested. The lack of correlation in clinicopathological parameters and patient demographics suggest that ISL1 and LHX5 may not be suitable prognostic markers for tumour progression because the expression is relatively high in a significant subset of tumours. Thus, these proteins may be potentially more useful as therapeutic targets against bladder cancer.

## Conclusion

In this study, we demonstrate that ISL1 expression increases with the stage of urothelial carcinoma of the bladder. Nevertheless, the expression is decreased in high grade urothelial carcinoma of the bladder. Although not distinctively differentially expressed, the confirmation that the expression of ISL1 and LHX5 is found in a high proportion of tumours across all tumour stages and grades suggest that both these proteins have significant roles during the multistep tumourigenesis of urothelial carcinoma of the bladder. Both proteins might be potential therapeutic targets against urothelial carcinoma. However, further functional studies of the protein need to be done to confirm the functional significance of the expression and role of ISL1 and LHX5 in urothelial carcinoma of the bladder.

## Figures and Tables

**Table 1:** Primary antibodies for immunohistochemical staining

Primary antibody	Species	Dilution	Incubation time	Supplier
ISL1 (AB86472)	Mouse monoclonal	1:2500	4 h	Abcam
LHX5 (AB88185)	Mouse monoclonal	1:8000	1 h	Abcam

**Table 2:** A semi-quantitative scoring system for immunohistochemical study

Percent positivity	Score	Staining intensity	Score
No positive tumour cells	0	No staining	0
Less than 10% positive tumour cells	1	Weak staining	1
10% to 50% positive tumour cells	2	Moderate staining	2
More than 50% positive tumour cells	3	Strong staining	3

Notes: Final score = Percent positivity × Staining intensity; Score: 0 = negative; ≤ 4 = low expression; ≥ 6 = high expression

**Table 3:** Correlation between immunohistochemical expression of ISL1 and LHX5 with demographic and clinicopathological parameters of urothelial carcinoma of the bladder

Characteristics	No. of cases (%)	ISL1 Expression			*P*^a^	LHX5 Expression			
	
		Negative *n* (%)	Low *n* (%)	High *n* (%)		Negative *n* (%)	Low *n* (%)	High *n* (%)	*P*^a^
Gender									
Male	92 (92)	6 (6.5)	24 (26.1)	62 (67.4)	0.637	2 (2.2)	21 (22.8)	69 (75.0)	0.910
Female	8 (8)	0 (0)	3 (37.5)	5 (62.5)		0 (0)	2 (25.0)	6 (75.0)	
Age (years)									
< 50	15 (15)	1 (6.7)	3 (20.0)	11 (73.3)	0.803	0 (0)	1 (6.7)	14 (93.3)	0.203
≥ 50	85 (85)	5 (5.9)	24 (28.2)	56 (65.9)		2 (2.3)	22 (25.9)	61 (71.8)	
Race									
Malay	59 (59)	4 (6.8)	14 (23.7)	41 (69.5)	0.858	2 (3.4)	10 (16.9)	47 (79.7)	0.285
Chinese	31 (31)	2 (6.5)	11 (35.5)	18 (58.0)		0 (0)	11 (35.5)	20 (64.5)	
Indian	5 (5)	0 (0)	1 (20.0)	4 (80.0)		0 (0)	2 (40.0)	3 (60.0)	
Others	5 (5)	0 (0)	1 (20.0)	4 (80.0)		0 (0)	0 (0)	5 (100)	
Stage									
Stage 0	21 (21)	0 (0)	7 (33.3)	14 (66.7)	0.886	1 (4.8)	4 (19.0)	16 (76.2)	0.476
Stage 1	21 (21)	1 (4.8)	7 (33.3)	13 (61.9)		1 (4.8)	3 (14.3)	17 (80.9)	
Stage 2	14 (14)	1 (7.1)	4 (28.6)	9 (64.3)		0 (0)	3 (21.4)	11 (78.6)	
Stage 3	13 (13)	1 (7.7)	3 (23.1)	9 (69.2)		0 (0)	6 (46.2)	7 (53.8)	
Stage 4	31 (31)	3 (9.7)	6 (19.4)	22 (70.9)		0 (0)	7 (22.6)	24 (77.4)	
Tumour invasion									
NMIBC	42 (42)	1 (2.4)	14 (33.3)	27 (64.3)	0.255	2 (4.8)	7 (16.7)	33 (78.5)	0.126
MIBC	58 (58)	5 (8.6)	13 (22.4)	40 (69.0)		0 (0)	16 (27.6)	42 (72.4)	
Grade									
Low grade^b^	25 (25)	0 (0)	6 (24.0)	19 (76.0)	0.248	2 (8.0)	6 (24.0)	17 (68.0)	0.044^a^
High grade^c^	75 (75)	6 (8.0)	21 (28.0)	48 (64.0)		0 (0)	17 (22.7)	58 (77.3)	
Lymph node metastasis									
Yes	25 (25)	2 (8.0)	5 (20.0)	18 (72.0)	0.621	0 (0)	6 (24.0)	19 (76.0)	0.710
No	75 (75)	4 (5.3)	22 (29.3)	49 (65.3)		2 (2.7)	17 (22.7)	56 (74.6)	
Metastasis									
Yes	23 (23)	3 (13.0)	4 (17.4)	16 (69.6)	0.172	0 (0)	6 (26.1)	17 (73.9)	0.696
No	77 (77)	3 (3.9)	23 (29.9)	51 (66.2)		2 (2.6)	17 (22.1)	58 (75.3)	

Notes:

a statistically significant (*P* < 0.05);

b Grade 1;

c Grade 2 and 3;

NMIBC = non-muscle invasive bladder cancer; MIBC = muscle invasive bladder cancer
